# Subpar reporting of pre‐analytical variables in RNA‐focused blood plasma studies

**DOI:** 10.1002/1878-0261.13647

**Published:** 2024-04-02

**Authors:** Céleste Van Der Schueren, Philippe Decruyenaere, Francisco Avila Cobos, Johanna Bult, Jill Deleu, Laudonia Lidia Dipalo, Hetty Hilde Helsmoortel, Eva Hulstaert, Annelien Morlion, Elena Ramos Varas, Kathleen Schoofs, Wim Trypsteen, Eveline Vanden Eynde, Hanne Van Droogenbroeck, Kimberly Verniers, Jo Vandesompele, Anneleen Decock

**Affiliations:** ^1^ Department of Biomolecular Medicine Ghent University Belgium; ^2^ Department of Hematology Ghent University Hospital Belgium; ^3^ OncoRNALab, Cancer Research Institute Ghent (CRIG) Belgium; ^4^ Department of Hematology University Medical Center Groningen The Netherlands; ^5^ Department of Dermatology AZ Sint‐Blasius Belgium; ^6^ Translational Oncogenomics and Bioinformatics Lab Cancer Research Institute Ghent (CRIG) Belgium; ^7^ Center for Medical Biotechnology VIB‐UGent Belgium; ^8^ CellCarta Belgium

**Keywords:** cell‐free RNA, circulating cell‐free RNA, extracellular RNA, liquid biopsies, plasma, pre‐analytical variable

## Abstract

Extracellular RNA (cell‐free RNA; exRNA) from blood‐derived liquid biopsies is an appealing, minimally invasive source of disease biomarkers. As pre‐analytical variables strongly influence exRNA measurements, their reporting is essential for meaningful interpretation and replication of results. The aim of this review was to chart to what extent pre‐analytical variables are documented, to pinpoint shortcomings and to improve future reporting. In total, 200 blood plasma exRNA studies published in 2018 or 2023 were reviewed for annotation of 22 variables associated with blood collection, plasma preparation, and RNA purification. Our results show that pre‐analytical variables are poorly documented, with only three out of 22 variables described in over half of the publications. The percentage of variables reported ranged from 4.6% to 54.6% (mean 24.84%) in 2023 and from 4.6% to 57.1% (mean 28.60%) in 2018. Recommendations and guidelines (i.e., BRISQ, ASCO‐CAP, BloodPAC, PPMPT, and CEN standards) have currently not resulted in improved reporting. In conclusion, our results highlight the lack of reporting pre‐analytical variables in exRNA studies and advocate for a consistent use of available standards, endorsed by funders and journals.

## Graphical abstract

Pre‐analytical variables strongly influence the analysis of extracellular RNA (cell‐free RNA; exRNA) derived from blood plasma. Their reporting is essential to allow interpretation and replication of results. By evaluating 200 exRNA studies, we pinpoint a lack of reporting pre‐analytical variables associated with blood collection, plasma preparation and RNA purification, and advocate for a consistent use of available reporting standards.

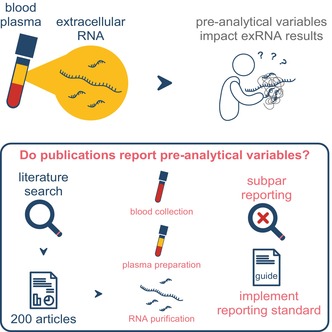

**Abbreviations**
ACD: acid‐citrate‐dextrose
ASCO‐CAP: American Society of Clinical Oncology‐College of American Pathologists
BRISQ: Biospecimen Reporting for Improved Study Quality
ccfDNA: circulating cell‐free DNA
ccfRNA: circulating cell‐free RNA
CEN: European Committee for Standardization
cfDNA: cell‐free DNA
cfRNA: cell‐free RNA
circRNA: circular RNA
CTC: circulating tumor cell
ctDNA: circulating tumor DNA
EDTA: ethylenediamine tetraacetic acid
ERCC: Extracellular RNA Communication Consortium
EV: extracellular vesicle
exRNA: extracellular RNA
lncRNA: long non‐coding RNA
miRNA: microRNA
mRNA: messenger RNA
MTDE: Minimum Technical Data Elements
NA: not applicable
PPMPT: Pre‐analytics for Precision Medicine Project Team
RCF: relative centrifugal force
rpm: revolutions per minute
snoRNA: small nucleolar RNA
snRNA: small nuclear RNA
tRF: transfer RNA‐derived fragments

## 1 Introduction

Blood‐derived liquid biopsies have the potential to revolutionize clinical practice, as they are minimally invasive, easy to obtain, and offer a wide range of analytes to be studied, including proteins, metabolites, blood cells, circulating tumor cells, extracellular vesicles (EVs), and extracellular nucleic acids. Together with their ability to reflect inter‐ and intra‐tumor heterogeneity, and the possibility of repeated measurements through longitudinal profiling during disease course or treatment make liquid biopsies an appealing source of biomarkers [1, 2]. Although most studies have focused on the use of cell‐free or circulating tumor DNA fragments (cfDNA/ctDNA), there has been increased interest in different forms of coding and non‐coding extracellular RNAs (exRNAs), both circulating and EV/platelet‐encapsulated, including messenger RNA (mRNA), microRNA (miRNA), long non‐coding RNA (lncRNA), and circular RNA (circRNA). Higher circulating levels have been found in patients with solid and hematological malignancies, where these are considered to play crucial roles in intercellular communication and to contribute to proliferation, malignant transformation, angiogenesis, immune response escape, and metastasis [1, 2]. Moreover, exRNAs are increasingly being exploited as biomarkers for diagnosis, disease phenotype, therapy response, and prognosis [3, 4, 5, 6, 7]. Besides malignant diseases, the detection of viral or bacterial RNA in blood has been used for non‐invasive diagnosis or monitoring of infectious diseases, and changes in the abundance of certain exRNAs have been shown to predict and detect coronary artery disease, myocardial infarction, stroke, and venous thromboembolism, as well as neurodegenerative disorders and psychiatric diseases [8, 9].

Although highly promising, the interpretation and replication of results on exRNA abundance in plasma is challenging, as the results of quantification technologies, including PCR and sequencing, are strongly impacted by pre‐analytical variables in sample collection, processing, and profiling (Appendix S1). For instance, previous research demonstrated that plasma concentrations of several circulating RNAs significantly decreased upon administration of heparin, a commonly used anticoagulant in the hospital setting, and that plasma processing conditions, as well as residual platelet levels substantially influenced the detection of circulating mRNAs and microRNAs [10, 11, 12]. Furthermore, the exRNAQC Consortium demonstrated that the choice of blood collection tube, time between draw and plasma preparation, and RNA purification method considerably impact mRNA and small RNA profiles [13]. Other consortia such as the NIH's Extracellular RNA Communication Consortium (ERCC), SPIDIA/SPIDIA4P (https://www.spidia.eu), BloodPAC, CANCER‐ID (https://www.imi.europa.eu/projects‐results/project‐factsheets/cancer‐id) and ELBS (http://www.elbs.eu), have been set up to evaluate the impact of pre‐analytical variables on exRNA research results, as well as to provide guidelines, aiming to standardize the research field [14, 15, 16, 17].

These collective efforts have clearly demonstrated that adequate reporting of experimental parameters in exRNA‐based studies is essential to critically interpret, compare, and replicate research results [4, 13, 18]. In order to evaluate to what extent these pre‐analytical variables are currently reported in literature, to analyze potential pitfalls, and to define future best practices, we performed an extensive and in‐depth review of a representative corpus of the current exRNA literature. Based on the available literature, a total of 22 pre‐analytical variables were identified and grouped in three main categories: blood collection, plasma preparation, and RNA purification process (Table 1). Lastly, to analyze the evolution in recent years, a period in which exRNA research has drastically increased, results obtained from articles published in 2023 were compared to those published in 2018.

**Table 1:** Reporting of 22 encoded pre‐analytical variables in the blood collection, plasma preparation or RNA purification process was evaluated in a representative corpus of 200 publications studying exRNA in blood plasma. A description for each of the variables is given. These variables are encoded as unknown (value 0), reported (value 1), reported in insufficient detail (value 2) or as not applicable to the publication (NA; see Appendix S3B). In the variable names, B stands for blood collection, P for plasma preparation and R for RNA purification. C stands for encoded variable. Raw data on the different variables (i.e., the corresponding BA, PA and RA variables) are available in Appendix S3A,C. Note that the data related to the individual centrifugation steps was summarized into a single variable (i.e. PC_centrifugation) for in‐depth analysis.

Variable	Description
Blood collection	
BC_fasting	Any info on the fasting status of the donor
BC_tube_anticoagulant	Anticoagulant of blood collection tube
BC_needle	Gauge number of needle
BC_tube_number	Number of collected blood collection tubes
BC_tube_order	Order in which multiple blood collection tubes are collected
BC_amount	Collected blood volume
BC_transport_time	Time interval between blood collection and arrival in lab
BC_transport_temperature	Temperature at which blood is transported
BC_blood_storage	Temperature at which blood is stored until further processing
Plasma preparation	
PC_time_interval	Time interval between blood collection and processing
PC_centrifugation	Variable collecting all info on number and details of centrifugation steps
PC_number_of_steps	Number of centrifugation steps
PC_step_x_speed	Centrifugation speed (evaluated for each of the centrifugation steps)
PC_step_x_duration	Centrifugation duration (evaluated for each of the centrifugation steps)
PC_step_x_temperature	Centrifugation temperature (evaluated for each of the centrifugation steps)
PC_step_x_storage	Instant centrifugation or after storage (evaluated for each of the centrifugation steps)
PC_plasma_QC	Methodology to assess plasma hemolysis
PC_plasma_storage	Temperature at which plasma is stored until further processing
RNA purification	
RC_RNA_purification	Indication if RNA purification is performed or not
RC_plasma_input	Plasma volume used for RNA purification
RC_method	RNA purification method
RC_plasma_fraction	Purification starting from extracellular vesicles (EVs) or total plasma
RC_extra_purification	Additional RNA purification method
RC_DNase	Time point of DNase treatment (during or after RNA purification)
RC_yield	Methodology to assess RNA yield
RC_RNA_storage	Temperature at which RNA is stored until further processing
RC_RNA_QC	Methodology to assess RNA quality

## 2 Methods

At the start of the study conducted by the exRNAQC Consortium [13], a first literature search was performed (on August 30, 2018) on Web of Science using the following keywords: TOPIC: (Plasma) AND TOPIC: (RNA). On August 30, 2023, a second search was performed using the same keywords, enabling to assess reporting evolutions over time. By evaluating the abstract, the 100 most recent publications studying exRNA in human plasma were selected in both 2018 and 2023. Studies without full‐text or not written in English, or exclusively focusing on the analysis of serum were excluded. For publications making use of both plasma and serum, only the pre‐analytical variables associated with the analysis of plasma‐derived exRNA were reviewed. A study article ID was assigned to each of the selected articles (A001–A200; Appendix S2). All publications were peer‐reviewed (for A055 by the journal's editorial team). Documentation on pre‐analytical variables grouped in three main categories (i.e., pre‐analytical variables in the blood collection, plasma preparation or RNA purification process (Appendix S3A)) was evaluated and converted to 22 encoded variables (Table 1) by at least two independent researchers. Results were compared and disagreements were solved by consensus (Appendix S3B). Variables were encoded as unknown (value 0), reported (value 1), reported in insufficient detail (value 2), or as not applicable to the publication (NA). Of each included publication, the main manuscript, and supplemental files were screened. Cross‐references to other publications or manufacturer's manuals were not evaluated. The percentages of publications reporting on specific pre‐analytical variables were calculated based on the total number of papers, excluding these annotated as not applicable for that variable.

## 3 Results

**Fig. 1:**
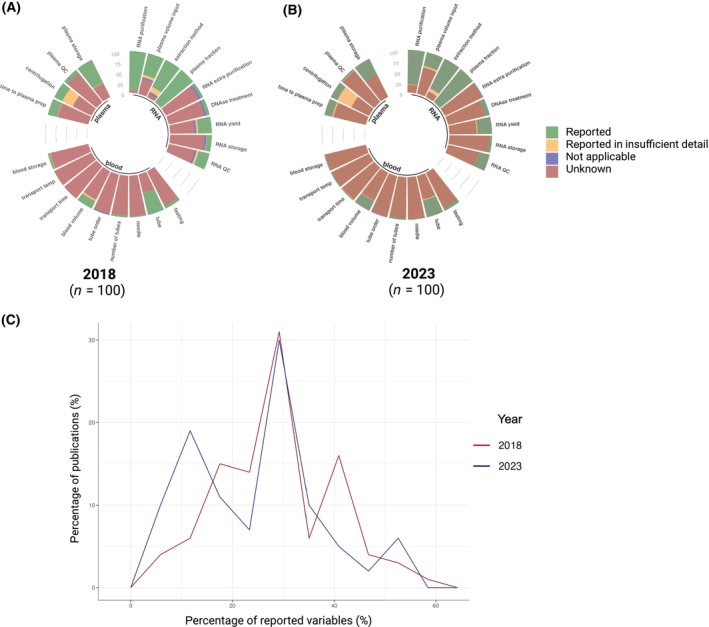
Pre‐analytical variables are poorly documented in exRNA studies. For a selection of 100 publications in 2018 (A) and 100 publications in 2023 (B) focussing on the analysis of exRNA in human blood plasma, information on 33 pre‐analytical variables in the blood collection, plasma preparation and RNA purification process was converted into 22 encoded variables (see Table 1 for variable explanation), annotated as unknown, reported, reported in insufficient detail or as not applicable to the publication. Panel (C) provides an overview of the percentage of variables that is reported within the publications of 2018 and 2023.

Figure 1 provides an overview of the results, demonstrating that pre‐analytical variables are currently very poorly documented in literature (panel B), with no improvement compared to 2018 (panel A and C). More precisely, only for three out of 22 variables (i.e., RC_RNA_purification, RC_method, and RC_plasma_fraction), the information is sufficiently detailed in more than half of the studied publications. Considering all variables, the percentage of variables that are adequately reported in each publication ranges from 4.6–54.6% (median of 27.3%). Below, further in‐depth results are described for each of the pre‐analytical steps preceding exRNA analysis.

### 3.1 Pre‐analytical variables associated with blood collection

Reporting of nine blood collection‐related pre‐analytical variables was studied. The anticoagulant in the blood collection tube (BC_tube_anticoagulant) was reported in 41.0% of the publications, whereby 90.24% used ethylenediamine tetraacetic acid (EDTA) tubes (Fig. 1 and Appendix S3A). Other anticoagulants used were sodium citrate and lithium heparin. In most publications, additional specifications on the type of blood collection tube are either lacking or no insightful information is provided. For example, only seven publications distinguish between the use of K2EDTA or K3EDTA (Appendix S3A), of which only two (A134 and A170) further specify the manufacturer's catalog number. The info ‘coagulant containing sterile tubes’ shared by A168 does not provide any useful information. Apart from the blood collection tube, other blood collection‐related variables (BC_fasting, BC_amount, BC_needle, BC_tube_number, BC_tube_order, BC_transport_time, BC_transport_temperature, and BC_blood_storage) are sparsely reported in literature, i.e., in 0–26.0% of the included studies (Fig. 1).

### 3.2 Pre‐analytical variables associated with plasma preparation

Reporting of pre‐analytical variables in the plasma preparation process was encoded into four variables: the time between blood draw and plasma processing (PC_time_interval), documentation of the centrifugation protocol (PC_centrifugation), the assessment of hemolysis (PC_plasma_QC), and the temperature at which the plasma is stored until further processing (PC_plasma_storage) (Fig. 1). Only 22.0% of the publications provided (incomplete) information on the time interval between blood collection and centrifugation (PC_time_interval). Indicated time intervals varied between ‘immediately’ and ‘within 24 h’, with 72.73% of publications reporting that blood was processed within 4 h after the blood draw (Appendix S3A). Documentation of the centrifugation protocol should include the number of centrifugation steps, as well as the speed, duration, and temperature of each of these steps, and whether centrifugation was instantly performed or upon biomaterial storage (freezing). Although approximately half of the publications report on these pre‐analytical variables, the information is often incomplete or unclear, resulting in absent or insufficient details in 83% of the studies (PC_centrifugation). For example, in 27.08% of the publications, centrifugation speed is indicated in revolutions per minute (rpm) instead of relative centrifugal force (RCF), the actual generated g‐force. In most publications that report the centrifugation protocol, single‐spun plasma is prepared (53.06%) as opposed to multiple‐spun plasma. Note that the centrifugation speed, duration, and temperature are highly variable across publications (Appendix S3A). Hemolysis (PC_plasma_QC) was objectively assessed in only 4.0% of the studies. Lastly, 42.0% of the publications indicates at which temperature the plasma is stored until further processing, of which the majority (95.24%) indicate that plasma is stored at −80 °C.

### 3.3 Pre‐analytical variables associated with RNA purification

For the RNA purification process, nine encoded variables were evaluated (Fig. 1). Remarkably, in 18% of the included publications, it is not clearly stated whether purification was performed or not (RC_RNA_purification). In total, 74% of the studies made use of total (neat) plasma, 22% of EVs, and 4% of both plasma and EV for exRNA purification (RA_plasma_fraction; Appendix S3A). The plasma input volume (RC_plasma_input) was only reported unambiguously in 33.0% of papers and ranged from 100 μL to 4.5 mL. Several publications only report plasma input volume ranges (A134) or use different volumes in distinct study groups (A147). The RNA purification method (RC_method) was among the most frequently annotated variables (77.0%). Notably, there is a high variance among the purification methods used and often incorrect/incomplete names of commercial products used for RNA purification (e.g., “A total RNA extraction kit (Qiagen, Hilden, Germany)”, A165; “miRNeasy kit; Qiagen”, A131) or vague descriptions (e.g., “an RNA extraction kit”, A156; “a proprietary method”, A141; “a phenol/chloroform isolation method”, A162/A169; “CircRNA extraction standard protocol”, A199) are given. Only few articles report on the performance of an extra RNA purification step (2.0%; RC_extra_purification) and/or DNase treatment (12.0%; RC_DNase). RNA yield (RC_yield), quality assessment (RC_RNA_QC), and storage (RC_RNA_storage) are adequately reported in 34.0%, 27.0%, and 8.0% of the studies, respectively. Of the publications that report on storage temperature, all of them specified that extracted exRNA is kept at −80 °C.

### 3.4 Trend in recent years

**Fig. 2:**
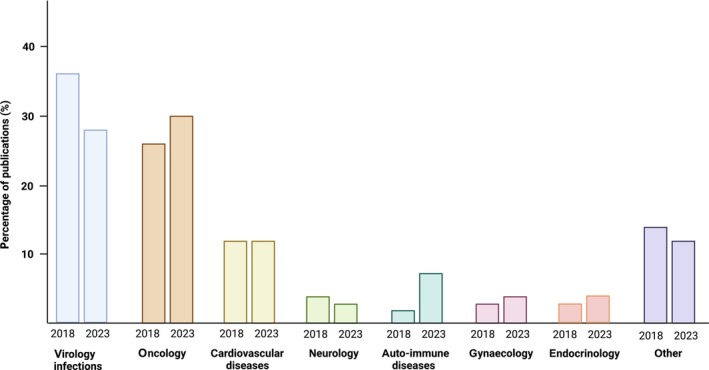
The research fields covered by the selected exRNA publications. This figure depicts the research fields that were most frequently covered within the included exRNA publications of 2018 and 2023.

Compared to 2018, no improvement is seen in reporting pre‐analytical variables with even decreased reporting values for four out of 22 variables: BC_tube_anticoagulant (41% in 2023 vs 55% in 2018), PC_plasma_storage (42% vs 61%), RC_plasma_input (33% vs 53%), RC_RNA_purification (81% vs 95%) (Fig. 1A,B). The mean percentages of variables that are adequately reported in each publication decreased from 28.60% in 2018 to 24.97% in 2023. Similar to 2018, the most used anticoagulant blood tube remained EDTA (92.7% and 90.24%, in 2018 and 2023, respectively). In the majority of studies, plasma is prepared within 4 h after blood draw (91.3% vs 72.73% in 2018 and 2023, respectively) with approximately half of the studies using a single‐step centrifugation (41.9% vs 53.06% in 2018 and 2023, respectively). The main fraction that was studied was total plasma (86.0% and 74%, respectively in 2018 and 2023), although an increasing number of studies have focused (also) on EVs (14.0% and 26.0%, respectively in 2018 and 2023). The most used RNA purification methods in 2018 were the miRNeasy Serum/Plasma kit (cat no. 217184; Qiagen) and the miRCURY RNA Isolation kit Biofluids (cat no. 300112; Exiqon A/S), applied in respectively 10% and 11% of the studies. In 2023, the most used method was the miRNeasy Serum/Plasma kit (cat no. 217184; Qiagen), applied in 15% of publications. Lastly, the research fields on which the publications focused were largely comparable between 2018 and 2023, although oncology‐related exRNA papers have surpassed papers on virology/infections (Fig. 2). The selected publications focus on different RNA biotypes, including microRNA, circular RNA (circRNA), messenger RNA (mRNA), long non‐coding RNA (lncRNA), transfer RNA‐derived fragments (tRF), small nucleolar RNA (snoRNA) and small nuclear RNA (snRNA).

## 4 Discussion

In the past decade, exRNA has increasingly been recognized as a valuable source of liquid biopsy biomarkers in a variety of malignant diseases, due to its disease‐specificity, its ability to reflect dynamic changes during disease course or treatment, and its possibility of longitudinal monitoring in a minimally invasive approach. Most errors in laboratory medicine occur in the extra‐analytical, especially in the pre‐analytical [19], phases of the testing process, and previous studies have shown that these variables have a major impact on exRNA profiling (Appendix S1). This review illustrates their poor documentation in a representative corpus of 200 publications focussing on exRNA analysis. The reporting of pre‐analytical variables was equally poor in both high‐ and lower impact journals (result not shown). Moreover, by comparing the results from 2023 to those obtained in 2018, we illustrate that there has been no improvement in reporting, despite ongoing efforts, evidenced by the development of several guidelines in recent years [14, 15, 16, 17]. The ensuing lack of standardization greatly hampers interpretation, replication, and validation of previous results, prerequisites for the development and clinical implementation of robust exRNA biomarkers.

In total, annotation on pre‐analytical variables associated with blood collection, plasma preparation or RNA purification was screened, and converted into 22 encoded variables, summarizing whether information is lacking (unknown), reported, reported in insufficient detail, or whether reporting on the variable is not relevant for the publication (not applicable, Appendix S3). Remarkably, only three out of 22 variables were adequately reported in more than half of the publications (i.e., whether RNA purification is performed, the method of RNA purification, and the plasma fraction used). These variables are frequently investigated in literature and as such a certain level of awareness on the importance of reporting these pre‐analytical variables exists within the exRNA research community. Concerning the plasma fraction, it may be worthwhile to highlight that serum and plasma represent different biofluid types, prepared using different blood collection tube types. These terms can, therefore, not be used interchangeably (as erroneously done in A088, A116, and A188). Besides these, however, other variables in all stages of the pre‐analytical process have been shown to significantly impact exRNA results but are currently rarely reported. During the blood collection phase, the choice of the blood collection tube anticoagulant, the fasting status of the patients, the needle used for blood draw, the tube order (if collecting multiple ones), the transport time and temperature, as well as the storing temperature of the blood tube all influence subsequent results [11, 13, 20, 21, 22, 23, 24, 25, 26, 27, 28, 29]. Similarly, in the plasma preparation phase, the time interval until plasma processing heavily affects the exRNA content [13, 29] and the assessment of hemolysis is critical, as contaminating hemoglobin can induce PCR inhibition, and the release of red blood cell RNA can change the exRNA profile [30, 31, 32]. Lastly, examples of pre‐analytical variables during exRNA purification include the use of DNase for gDNA removal, which is crucial for certain exRNA analyses [13, 33], as well as the plasma volume input for purification [13]. Moreover, if mentioned, crucial details on the used blood collection tube or RNA purification method are often missing, such as the manufacturer's product name and catalog number. General terms such as “plasma collected using standard procedures” (in A026) or “tubes of regular type” (in A007) should be avoided. Of note, some publications explicitly state the inability to share information on pre‐analytical variables for various reasons, including privacy reasons (A003). Although researchers need to adhere to legal obligations and ethical standards, the procedure of (pseudo)anonymization enables adequate reporting of pre‐analytical variables in many cases while guaranteeing the subjects' privacy. Finally, we would recommend to avoid to refer to manufacturer's manuals or other publications for detailed pre‐analytical variable descriptions. These sources of information are often incomplete, or even provide conflicting information compared to the information available in the publication at hand (for example for A056).

The American Society of Clinical Oncology (ASCO)/College of American Pathologists (CAP) HER2 testing guideline for breast cancer (2007) was the first evidence‐based guideline that directly addressed the necessity of controlling pre‐analytical variables for biospecimens in cancer biomarker testing [34]. In 2011, the recommendations on Biospecimen Reporting for Improved Study Quality (BRISQ) were published, describing the pre‐analytical variables that should be reported when human biospecimens are used with the goal to help evaluate, interpret, and compare, and reproduce experimental results [30]. In recent years, several guidelines were developed for liquid biopsy studies that advocate for standardized reporting (Table 2) [17, 30, 35, 36]. Recently, these efforts culminated in multiple CEN (European Committee for Standardization) technical pre‐analytical standards, which have emerged from the SPIDIA4P program [37]. Separate standards have been published for circulating cell‐free DNA (ccfDNA; EN ISO 20186‐3:2019), circulating cell‐free RNA (ccfRNA; CEN/TS 17742:2022), circulating tumor cells (CTCs; CEN/TS 17390:2020) and EVs (CEN/TS 17747:2022), illustrating the importance of omic‐specific guidelines for liquid biopsy studies (https://standards.cen.eu). The ccfRNA standard represents the most comprehensive overview of variables that should be considered and reported when analyzing exRNA (Table 2) [38]. The development of increasingly large networks of collaborating facilities will further underline the importance of these efforts and the need of standardization [39]. Moreover, in the coming years, this field will be in ongoing evolution, as future benchmarking studies will provide additional insights and guide evidence‐based choices within pre‐analytical variables. Based on the knowledge gained from these studies and the currently available reporting guidelines, specific recommendations for exRNA‐based studies should be developed. For this purpose, we strongly urge the research community to adhere to currently available guidelines by consistently and unambiguously reporting pre‐analytical variables, which will prove essential in guiding the rapidly increasing exRNA research field, in optimizing workflows, and in facilitating future clinical implementation of results in this era of precision medicine.

**Table 2:** Recommendations of previous guidelines on the reporting of pre‐analytical variables in liquid biopsy research. ASCO‐CAP, American Society of Clinical Oncology (ASCO)‐College of American Pathologists (CAP); BloodPAC, The Blood Profiling Atlas in Cancer; BRISQ, recommendations on Biospecimen Reporting for Improved Study Quality; PPMPT, Pre‐analytics for Precision Medicine Project Team.

Pre‐analytical variable	BRISQ^a^ [30]	ASCO‐CAP^b^ [36]	PPMPT^c^ [35]	BloodPAC^d^ [17]	CEN/TS 17742:2022^e^ [38]
Sample characteristics					
Biospecimen site	×	×			
Anatomical site	×	×			
Composition assessment	×			×	
Patient characteristics					
Disease status	×	×			×
Clinical characteristics	×	×			×
Vital state (alive/death)	×	×			×
Clinical diagnosis	×	×			×
Pathology diagnosis	×	×			×
Lifestyle parameters		×			×
Blood collection					
Conditions prior to collection (e.g. fasting status/anesthetics)					×
Blood tube	×	×	×	×	×
Tube fill/inversion		×	×		×
Tube draw order		×	×		
Type of stabilization	×	×	×		×
Blood transport conditions		×		×	×
Plasma preparation					
Blood fractionalization method		×	×	×	×
Time to fractionation		×	×	×	×
Preservation of plasma					
Time to freezer				×	×
Type of long‐term preservation	×		×		×
Constitution of preservative	×				×
Storage temperature	×	×	×	×	×
Storage duration	×				×
Shipping temperature	×	×		×	×
Freeze–thaw cycles		×	×		×
Analyte isolation					
Analyte isolation method		×		×	×
DNase treatment					×
Analyte yield				×	×
Analyte purity					×
Analyte preservation					
Type of storage vessel					×
Storage temperature		×			×
Storage duration		×			×
Freeze–thaw cycles					×
Assay					
Sample selection	×				
Time to assay				×	
Assay method		×		×	

^a^ Tier 1, variables recommended to report according to BRISQ. These guidelines are intended for any human biospecimen.

^b^ ASCO‐CAP guidelines are originally intended for circulating tumor DNA specimens.

^c^ PPMPT guidelines are originally intended for tissue and blood specimens.

^d^ Minimum Technical Data Elements (MTDEs) according to BloodPAC. These guidelines are originally intended for cell‐free DNA specimens.

^e^ The CEN/TS 17742:2022 standard is specifically intended for exRNA specimens.

## 5 Conclusions

The impact of pre‐analytical variables on subsequent exRNA analyses has been increasingly recognized, as evidenced by the development of multiple guidelines and a European standard. Adequate and standardized reporting is a prerequisite to allow meaningful interpretation, critical comparison, replication and validation of experimental design and research results. Despite these ongoing efforts, our results highlight the persistent lack of their documentation in exRNA studies and strongly advocate for their consistent reporting.

## Conflict of interest

JV is consultant at CellCarta, a global CRO providing RNA quantification services. The other authors have no conflicts of interest to declare.

## Author contributions

CVDS, PD, HHH, AD, and JV contributed to conceptualization; CVDS and AD contributed to methodology and validation; CVDS, PD, HHH, and AD contributed to formal analysis; CVDS, PD, FAC, JD, EH, AM, KS, EVE, KV, JB, LLD, ERV, HVD, WT, KS, and AD contributed to investigation and data curation; CVDS contributed to resources; CVDS, PD, and AD contributed to writing—original draft; PD, AD, and JV contributed to writing—review & editing; PD contributed to visualization; JV contributed to supervision; CVDS, HHH, and AD contributed to project administration.

## Supporting information


**Appendix S1.** ExRNA analysis is strongly determined by pre‐analytical variables in sample collection, processing, and profiling. Pre‐analytical variables impacting exRNA quantification are listed, including literature references. Note that the available literature on these pre‐analytics may not be limited to the references indicated in the overview.

**Appendix S2.** In total, 200 publications studying extracellular RNA in human plasma were selected. Shown are a reference list of the selected publications and corresponding study article IDs.

**Appendix S3.** In total, reporting of pre‐analytical variables was encoded into 22 variables. A&C. Raw data. For each publication (Article ID), the information on the pre‐analytical variables as reported in the original publication is shown. Empty cells indicate that no information is available. B&D. Encoded data. The raw data is encoded into 22 variables (see Table 1 for variable explanation). Variables were encoded as unknown (value 0), reported (value 1), reported in insufficient detail (value 2), or as not applicable to the publication (NA). In A‐D, columns indicated in yellow show raw or encoded data of the individual centrifugation steps. These columns were summarized into a single variable (i.e. PC_centrifugation) for in‐depth analysis.
