# Supplementary material for: Subpar reporting of pre‐analytical variables in RNA‐focused blood plasma studies
Source: Mol Oncol. 2024 Apr 2;19(7):1968–78. doi: 10.1002/1878-0261.13647 (PMC12234387; doi:10.1002/1878-0261.13647)
Supplement: Supplementary file 1 — Appendix S1. ExRNA analysis is strongly determined by pre‐analytical variables in sample collection, processing, and profiling. Pre‐analytical variables impacting exRNA quantification are listed, including literature references. Note that the available literature on these pre‐analytics may not be limited to the references indicated in the overview. [file MOL2-19-1968-s001.docx]

**Table 1. Non-exhaustive list of pre-analytical variables that impact the extracellular RNA profile in blood plasma**

| **pre-analytical variable** | **references** |
| --- | --- |
| fasting status of the subject | (1,2) |
| needle for blood draw | (3–5) |
| blood collection tube anticoagulant | (6–8) |
| blood collection tube order | (9,10) |
| transport temperature | (11) |
| blood storage | (11,12) |
| time interval between blood draw and processing | (6,12) |
| centrifugation speed for plasma preparation | (13) |
| centrifugation duration for plasma preparation | (13) |
| centrifugation temperature for plasma preparation | (13) |
| plasma QC | (14,15) |
| plasma storage | (16) |
| plasma fraction for RNA purification | (17) |
| plasma input for RNA purification | (6) |
| RNA purification method | (6) |
| DNase treatment of exRNA | (18) |
| extra RNA purification | (6) |
| RNA QC | (19) |

**References**

1. Daimiel L, Micó V, Valls RM, et al. Impact of phenol-enriched virgin olive oils on the postprandial levels of circulating microRNAs related to cardiovascular disease. Mol Nutr Food Res 2020;64(15):e2000049; doi: 10.1002/mnfr.202000049.
2. Quintanilha BJ, Pinto Ferreira LR, Ferreira FM, et al. Circulating plasma microRNAs dysregulation and metabolic endotoxemia induced by a high-fat high-saturated diet. Clin Nutr 2020;39(2):554–562; doi: 10.1016/j.clnu.2019.02.042.
3. Lippi G, Salvagno GL, Brocco G, et al. Preanalytical variability in laboratory testing: Influence of the blood drawing technique. Clin Chem Lab Med 2005;43(3):319–325; doi: 10.1515/CCLM.2005.055.
4. Lippi G, Salvagno GL, Montagnana M, et al. Influence of the needle bore size on platelet count and routine coagulation testing. Blood Coagul Fibrinolysis 2006;17(7):557–561; doi: 10.1097/01.mbc.0000245300.10387.ca.
5. Cheng HH, Yi HS, Kim Y, et al. Plasma processing conditions substantially influence circulating microRNA biomarker levels. PLoS One 2013;8(6):e64795; doi: 10.1371/journal.pone.0064795.
6. ExRNAQC Consortium. Performance of RNA purification kits and blood collection tubes in the Extracellular RNA Quality Control (exRNAQC) study. bioRxiv; doi: 10.1101/2021.05.11.442610.
7. Boeckel J, Thomé CE, Leistner D, et al. Heparin selectively affects the quantification of microRNAs in human blood samples. Clin Chem 2013;59(7):1125–1127; doi: 10.1373/clinchem.2012.199505.
8. Zhelankin AV, Iulmetova LN, Sharova EI. The impact of the anticoagulant type in blood collection tubes on circulating extracellular plasma microRNA profiles revealed by small RNA Sequencing. Int J Mol Sci 2022;23(18):10340; doi: 10.3390/ijms231810340.
9. Bazzano G, Galazzi A, Giusti GD, et al. The order of draw during blood collection: A systematic literature review. Int J Environ Res Public Health 2021;18(4):1568; doi: 10.3390/ijerph18041568.
10. Ercan Ş, Ramadan B, Gerenli O. Order of draw of blood samples affect potassium results without K-EDTA contamination during routine workflow. Biochem Med (Zagreb) 2021;31(2):020704; doi: 10.11613/BM.2021.020704.
11. Malentacchi F, Pizzamiglio S, Wyrich R, et al. Effects of transport and storage conditions on gene expression in blood samples. Biopreserv Biobank 2016;14(2):122–128; doi: 10.1089/bio.2015.0037.
12. Sun J, Yang X, Wang T, et al. Evaluating the effects of storage conditions on multiple cell-free RNAs in plasma by high-throughput sequencing. Biopreserv Biobank 2022; doi: 10.1089/bio.2022.0004.
13. Sorber L, Zwaenepoel K, Jacobs J, et al. Circulating cell-free DNA and RNA analysis as liquid biopsy: optimal centrifugation protocol. Cancers (Basel) 2019;11(4):458; doi: 10.3390/cancers11040458.
14. Smith MD, Leemaqz SY, Jankovic-karasoulos T, et al. Haemolysis detection in microRNA-seq from clinical plasma samples. Genes (Basel) 2022;13(7):1288; doi: 10.3390/genes13071288.
15. Kirschner MB, Kao SC, Edelman JJ, et al. Haemolysis during sample preparation alters microRNA content of plasma. PLoS One 2011;6(9):e24145; doi: 10.1371/journal.pone.0024145.
16. José M, Curtu S, Gajardo R, et al. The effect of storage at different temperatures on the stability of Hepatitis C virus RNA in plasma samples. Biologicals 2003;31(1):1–8; doi: 10.1016/s1045-1056(02)00067-2.
17. Jia J, Yang S, Huang J, et al. Distinct extracellular RNA profiles in different plasma components. Front Genet 2021;12:564780; doi: 10.3389/fgene.2021.564780.
18. Verwilt J, Trypsteen W, Van Paemel R, et al. When DNA gets in the way: a cautionary note for DNA contamination in extracellular RNA-seq studies. Proc Natl Acad Sci USA 2020;117(32):18934–18936; doi: 10.1073/pnas.2001675117.
19. Schuierer S, Carbone W, Knehr J, et al. A comprehensive assessment of RNA-seq protocols for degraded and low-quantity samples. BMC Genomics 2017;18(1):442; doi: 10.1186/s12864-017-3827-y.
