# Supplementary material for: Subpar reporting of pre‐analytical variables in RNA‐focused blood plasma studies
Source: Mol Oncol. 2024 Apr 2;19(7):1968–78. doi: 10.1002/1878-0261.13647 (PMC12234387; doi:10.1002/1878-0261.13647)
Supplement: Supplementary file 2 — Appendix S2. In total, 200 publications studying extracellular RNA in human plasma were selected. Shown are a reference list of the selected publications and corresponding study article IDs. [file MOL2-19-1968-s003.pdf]

A001. Bulgakova et al. miR-19 in blood plasma reflects lung cancer occurrence but is not specifically associated with radon exposure. Oncology Letters, 2018.

A002. Qureshi et al. δ-tocotrienol feeding modulates gene expression of EIF2, mTOR, protein ubiquitination through multiple-signaling pathways in chronic hepatitis C patients. Lipids in Health and Disease, 2018.

A003. Zhang et al. Plasma circular RNAs, hsa_circRNA_025016, predict postoperative atrial fibrillation after isolated off-pump coronary artery bypass grafting. Journal of the American Heart Association, 2018.

A004. Abravanel et al. A fully automated system using transcription-mediated amplification for the molecular diagnosis of hepatitis E virus in human blood and faeces. Journal of Clinical Virology, 2018.

A005. Zhou et al. Hepatitis E virus infection in HIV-infected patients: A large cohort study in Yunnan province, China. Journal of Medical Virology, 2018.

A006. Zhou et al. PLAUR confers resistance to gefitinib through EGFR/P-AKT/Survivin signaling pathway. Cellular Physiology and Biochemistry, 2018.

A007. Wu et al. Integrated assessment of differentially expressed plasma microRNAs in subtypes of nonsyndromic orofacial clefts. Medicine, 2018.

A008. Wang et al. Extracellular mRNA detected by molecular beacons in tethered lipoplex nanoparticles for diagnosis of human hepatocellular carcinoma. Plos One, 2018.

A009. Wang et al. RNase H2-dependent polymerase chain reaction and elimination of confounders in sample collection, storage, and analysis strengthen evidence that microRNAs in bovine milk are bioavailable in humans. Journal of Nutrition, 2018.

A010. Wang et al. A novel human pegivirus, HPgV-2 (HHpgV-1), is tightly associated with hepatitis C virus (HCV) infection and HCV/human immunodeficiency virus type 1 coinfection. Clinical Infectious Diseases, 2018.

A011. Taiwo et al. ACTG A5353: A pilot study of dolutegravir plus lamivudine for initial treatment of human immunodeficiency virus-1 (HIV-1)-infected participants with HIV-1 RNA < 500 000 copies/mL. Clinical Infectious Diseases, 2018.

A012. Susluer et al. Analysis of long non-coding RNA (lncRNA) expression in hepatitis B patients. Bosnian Journal of Basic Medical Sciences, 2018.

A013. Stranska et al. Comparison of membrane affinity-based method with size-exclusion chromatography for isolation of exosome-like vesicles from human plasma. Journal of Translational Medicine, 2018.

A014. Skardasi et al. Authentic patient-derived hepatitis C virus infects and productively replicates in primary CD4+ and CD8+ T lymphocytes in vitro. Journal of Virology, 2018.

A015. Shah et al. MicroRNAs associated with reverse left ventricular remodeling in humans identify pathways of heart failure progression. Circulation. Heart Failure, 2018.

A016. Salvi et al. Exosome-delivered microRNAs promote IFN-α secretion by human plasmacytoid DCs via TLR7. JCI Insight, 2018.

A017. Bao et al. Extracellular vesicle RNA sequencing reveals dramatic transcriptomic alterations between metastatic and primary osteosarcoma in a liquid biopsy approach. Annals of Surgical Oncology, 2018.

A018. Agiannitopoulos et al. Expression of miR-208b and miR-499 in Greek patients with acute myocardial infarction. In Vivo, 2018.

A019. Kinyua et al. Antiretroviral resistance among HIV-1 patients on first-line therapy attending a comprehensive care clinic in Kenyatta National Hospital, Kenya: A retrospective analysis. Pan African Medical Journal, 2018.

A020. Barzon et al. Virus and antibody dynamics in travelers with acute Zika virus infection. Clinical Infectious Diseases, 2018.

A021. Blissenbach et al. Hypoxia-induced changes in plasma micro-RNAs correlate with pulmonary artery pressure at high altitude. American Journal of Physiology. Lung Cellular and Molecular Physiology, 2018.

A022. Bokharaei-Salim et al. Investigation of the effects of a prevention of mother-to-child HIV transmission program among Iranian neonates. Archives of Virology, 2018.

A023. Rubio et al. Circulating miRNAs, isomiRs and small RNA clusters in human plasma and breast milk. Plos One, 2018.

A024. Ramos et al. Specific circulating microRNAs display dose-dependent responses to variable intensity and duration of endurance exercise. American Journal of Physiology. Heart and Circulatory Physiology, 2018.

A025. Max et al. Human plasma and serum extracellular small RNA reference profiles and their clinical utility. Proceedings of the National Academy of Sciences of the United States of America, 2018.

A026. Xiao-Long et al. Circular RNA circ_HIPK3 is down-regulated and suppresses cell proliferation, migration and invasion in osteosarcoma. Journal of Cancer, 2018.

A027. Sun et al. Long noncoding RNA SNHG5 is up-regulated and serves as a potential prognostic biomarker in acute myeloid leukemia. European Review for Medical and Pharmacological Sciences, 2018.

A028. Li et al. Circular RNA IARS (circ-IARS) secreted by pancreatic cancer cells and located within exosomes regulates endothelial monolayer permeability to promote tumor metastasis. Journal of Experimental & Clinical Cancer Research, 2018.

A029. Koupenova et al. Micro RNAs from DNA viruses are found widely in plasma in a large observational human population. Scientific Reports, 2018.

A030. Jumare et al. Cognitive function among antiretroviral treatment-naive individuals infected with human immunodeficiency virus type 1 subtype G versus CRF02_AG in Nigeria. Clinical Infectious Diseases, 2018.

A031. Ikoma et al. KSHV oral shedding and plasma viremia result in significant changes in the extracellular tumorigenic miRNA expression profile in individuals infected with the malaria parasite. Plos One, 2018.

A032. Heintz-Buschart et al. Small RNA profiling of low biomass samples: Identification and removal of contaminants. BMC Biology, 2018.

A033. Cheng et al. Circulating microRNAs and treatment response in the Phase II SWOG S0925 study for patients with new metastatic hormone-sensitive prostate cancer. The Prostate, 2018.

A034. Gandhi et al. Cumulative antiretroviral exposure measured in hair is not associated with measures of HIV persistence or inflammation among individuals on suppressive ART. Journal of Infectious Diseases, 2018.

A035. Franco et al. Large-scale screening of circulating microRNAs in individuals with HIV-1 mono-infections reveals specific liver damage signatures. Antiviral Research, 2018.

A036. Ferrero et al. Small non-coding RNA profiling in human biofluids and surrogate tissues from healthy individuals: Description of the diverse and most represented species. Oncotarget, 2018.

A037. Ferreira et al. Serological and molecular markers of hepatitis E virus infection in HIV-infected patients in Brazil. Archives of Virology, 2018.

A038. Dravid et al. Discordant CSF/plasma HIV-1 RNA in individuals on virologically suppressive antiretroviral therapy in Western India. Medicine, 2018.

A039. Dehghani-Dehej et al. Presence of different hepatitis C virus genotypes in plasma and peripheral blood mononuclear cell samples of Iranian patients with HIV infection. Journal of Medical Virology, 2018.

A040. de Jong et al. Epidemiology of sepsis-like illness in young infants: Major role of enterovirus and human parechovirus. Pediatric Infectious Disease Journal, 2018.

A041. Alidjinou et al. Enteroviruses in blood of patients with type 1 diabetes detected by integrated cell culture and reverse transcription quantitative real-time PCR. Acta Diabetologica, 2017.

A042. Belongie et al. Identification of novel biomarkers to monitor β-cell function and enable early detection of type 2 diabetes risk. Plos One, 2017.

A043. Ceolotto et al. miR-30c-5p regulates macrophage-mediated inflammation and pro-atherosclerosis pathways. Cardiovascular Research, 2017.

A044. Chéret et al. Impact of early cART on HIV blood and semen compartments at the time of primary infection. Plos One, 2017.

A045. Cornelissen et al. From clinical sample to complete genome: Comparing methods for the extraction of HIV-1 RNA for high-throughput deep sequencing. Virus Research, 2017.

A046. Dlouha et al. Analysis of circulating miRNAs in patients with familial hypercholesterolaemia treated by LDL/Lp(a) apheresis. Atherosclerosis. Supplements, 2017.

A047. Endzeliņš et al. Detection of circulating miRNAs: Comparative analysis of extracellular vesicle-incorporated miRNAs and cell-free miRNAs in whole plasma of prostate cancer patients. BMC Cancer, 2017.

A048. Etta et al. High level of HIV-1 drug resistance mutations in patients with unsuppressed viral loads in rural northern South Africa. AIDS Research and Therapy, 2017.

A049. Foye et al. Comparison of miRNA quantitation by Nanostring in serum and plasma samples. Plos One, 2017.

A050. Gao et al. MicroRNA-155, induced by FOXP3 through transcriptional repression of BRCA1, is associated with tumor initiation in human breast cancer. Oncotarget, 2017.

A051. He et al. Differential expression of long non-coding RNAs in patients with tuberculosis infection. Tuberculosis, 2017.

A052. Henrich et al. HIV-1 persistence following extremely early initiation of antiretroviral therapy (ART) during acute HIV-1 infection: An observational study. Plos Medicine, 2017.

A053. Huang et al. Circular RNA hsa_circ_0000745 may serve as a diagnostic marker for gastric cancer. World Journal of Gastroenterology, 2017.

A054. Jagdagsuren et al. The second molecular epidemiological study of HIV infection in Mongolia between 2010 and 2016. Plos One, 2017.

A055. Kalinina et al. The occurrence of the markers of hepatitis C among practically healthy residents of the Republic of Guinea: A pilot sutdy. Russian Journal of Infection and Immunity, 2017.

A056. Li et al. Modified high-throughput quantification of plasma microRNAs in heparinized patients with coronary artery disease using heparinase. Biochemical and Biophysical Research Communications, 2017.

A057. Nadeem et al. Clinicopathological features associated to miRNA-195 expression in patients with breast cancer: Evidence of a potential biomarker. Pakistan Journal of Medical Sciences, 2017.

A058. Nasrallah et al. Seroprevalence of hepatitis E virus among blood donors in Qatar (2013-2016). Transfusion, 2017.

A059. Ntelios et al. Elevated plasma levels of miR-29a are associated with hemolysis in patients with hypertrophic cardiomyopathy. Clinica Chimica Acta, 2017.

A060. Pan et al. Circular RNAs promote TRPM3 expression by inhibiting hsa-miR-130a-3p in coronary artery disease patients. Oncotarget, 2017.

A061. Perge et al. Evaluation and diagnostic potential of circulating extracellular vesicle-associated microRNAs in adrenocortical tumors. Scientific Reports, 2017.

A062. Pezuk et al. Measuring plasma levels of three microRNAs can improve the accuracy for identification of malignant breast lesions in women with BI-RADS 4 mammography. Oncotarget, 2017.

A063. Rivera-Barahona et al. Dysregulated miRNAs and their pathogenic implications for the neurometabolic disease propionic acidemia. Scientific Reports, 2017.

A064. Rossini et al. Comparison of Zika virus (ZIKV) RNA detection in plasma, whole blood and urine - Case series of travel-associated ZIKV infection imported to Italy, 2016. Journal of Infection, 2017.

A065. Salomon et al. Placental exosomes as early biomarker of preeclampsia: Potential role of exosomal microRNAs across gestation. Journal of Clinical Endocrinology & Metabolism, 2017.

A066. Sandoval-Bórquez et al. MicroRNA-335-5p is a potential suppressor of metastasis and invasion in gastric cancer. Clinical Epigenetics, 2017.

A067. Sebastiani et al. Circulating microRNA (miRNA) expression profiling in plasma of patients with gestational diabetes mellitus reveals upregulation of miRNA mir-330-3p. Frontiers in Endocrinology, 2017.

A068. Sheinerman et al. Circulating brain-enriched microRNAs as novel biomarkers for detection and differentiation of neurodegenerative diseases. Alzheimer's Research & Therapy, 2017.

A069. Borena et al. No molecular or serological evidence of Zikavirus infection among healthy blood donors living in or travelling to regions where Aedes albopictus circulates. Plos One, 2017.

A070. Hu et al. Circulating miR-125b but not miR-125a correlates with acute exacerbations of chronic obstructive pulmonary disease and the expressions of inflammatory cytokines. Medicine, 2017.

A071. Pan et al. Simultaneously monitoring immune response and microbial infections during pregnancy through plasma cfRNA sequencing. Clinical Chemistry, 2017.

A072. Li et al. Identification of circulating long noncoding RNA HOTAIR as a novel biomarker for diagnosis and monitoring of non-small cell lung cancer. Technology in Cancer Research & Treatment, 2017.

A073. Li et al. A plasma mir-125a-5p as a novel biomarker for Kawasaki disease and induces apoptosis in HUVECs. Plos One, 2017.

A074. McArdle et al. TORNADO-Theranostic One-Step RNA Detector; microfluidic disc for the direct detection of microRNA-134 in plasma and cerebrospinal fluid. Scientific Reports, 2017.

A075. Palich et al. Ebola virus RNA detection on fomites in close proximity to confirmed Ebola patients; N'Zerekore, Guinea, 2015. Plos One, 2017.

A076. Zhang et al. Plasma exosomes from HLA-sensitized kidney transplant recipients contain mRNA transcripts which predict development of antibody-mediated rejection. Transplantation, 2017.

A077. Tiedt et al. RNA-seq identifies circulating miR-125a-5p, miR-125b-5p, and miR-143-3p as potential biomarkers for acute ischemic stroke. Circulation Research, 2017.

A078. Meng et al. Screening and validation of differentially expressed extracellular miRNAs in acute pancreatitis. Molecular Medicine Reports, 2017.

A079. Sheinerman et al. Circulating brain-enriched microRNAs as novel biomarkers for detection and differentiation of neurodegenerative diseases. Alzheimer's Research & Therapy, 2017.

A080. Suzuki et al. Comprehensive detection of viruses in pediatric patients with acute liver failure using next-generation sequencing. Journal of Clinical Virology, 2017.

A081. Tan et al. Identification of circulating long non-coding RNA GAS5 as a potential biomarker for non-small cell lung cancer diagnosis. International Journal of Oncology, 2017.

A082. Xu et al. Plasma exosome miR-196a and miR-1246 are potential indicators of localized pancreatic cancer. Oncotarget, 2017.

A083. Xuan et al. Circulating long non-coding RNAs NRON and MHRT as novel predictive biomarkers of heart failure. Journal of Cellular and Molecular Medicine, 2017.

A084. Yang et al. Circular RNA circ-LDLRAD3 as a biomarker in diagnosis of pancreatic cancer. World Journal of Gastroenterology, 2017.

A085. Zhang et al. Identification and characterization of circular RNAs as a new class of putative biomarkers in diabetes retinopathy. Investigative Ophthalmology & Visual Science, 2017.

A086. Zhao et al. Circulating microRNA-34 family low expression correlates with poor prognosis in patients with non-small cell lung cancer. Journal of Thoracic Disease, 2017.

A087. Zhou et al. Clinical verification of plasma messenger RNA as novel noninvasive biomarker identified through bioinformatics analysis for lung cancer. Oncotarget, 2017.

A088. Alehagen et al. Significant changes in circulating microRNA by dietary supplementation of selenium and coenzyme Q10 in healthy elderly males. A subgroup analysis of a prospective randomized double-blind placebo-controlled trial among elderly Swedish citizens. Plos One, 2017.

A089. Chang et al. Relationship of human immunodeficiency virus viral load in cerebrospinal fluid and plasma in patients co-infected with cryptococcal meningitis. Open Forum Infectious Diseases, 2017.

A090. Del Re et al. The detection of androgen receptor splice variant 7 in plasma-derived exosomal RNA strongly predicts resistance to hormonal therapy in metastatic prostate cancer patients. European Urology, 2017.

A091. Elhamamsy et al. Circulating miR-92a, miR-143 and miR-342 in plasma are novel potential biomarkers for acute myeloid leukemia. International Journal of Molecular and Cellular Medicine, 2017.

A092. García-Olmo et al. Potential clinical significance of perioperative levels of mRNA in plasma from patients with cancer of the larynx or hypopharynx. Head & Neck, 2017.

A093. Ke et al. The combination of circulating long noncoding RNAs AK001058, INHBA-AS1, MIR4435-2HG, and CEBPA-AS1 fragments in plasma serve as diagnostic markers for gastric cancer. Oncotarget, 2017.

A094. Noroozi et al. The Effects of IFN-β 1a on the expression of inflammasomes and apoptosis-associated speck-like proteins in multiple sclerosis patients. Molecular Neurobiology, 2017.

A095. Ting et al. Quantification of BCR-ABL transcripts in peripheral blood cells and plasma of chronic myeloid leukemia patients at different stages of tyrosine kinase inhibitor treatment response. Tropical Journal of Pharmaceutical Research, 2017.

A096. Williamson et al. First cases of Zika virus-infected US blood donors outside states with areas of active transmission. Transfusion, 2017.

A097. Winckelmann et al. Romidepsin-induced HIV-1 viremia during effective antiretroviral therapy contains identical viral sequences with few deleterious mutations. Aids, 2017.

A098. Yeri et al. Total extracellular small RNA profiles from plasma, saliva, and urine of healthy subjects. Scientific Reports, 2017.

A099. Zhao et al. Plasma microRNA signature predicting weight gain among Mexican-American women. Obesity, 2017.

A100. Lagatie et al. Plasma-derived parasitic microRNAs have insufficient concentrations to be used as diagnostic biomarker for detection of Onchocerca volvulus infection or treatment monitoring using LNA-based RT-qPCR. Parasitology Research, 2017.

A101. Dunlop et al. L1CAM immunocapture generates a unique extracellular vesicle population with a reproducible miRNA fingerprint. RNA Biology, 2023.

A102. Luo et al. Hsa_circ_0044235 and hsa_circ_0001947 as novel biomarkers in plasma of patients with new-onset systemic lupus erythematosus. Journal of Immunotoxicology, 2023.

A103. Wang et al. Differences in drug resistance of HIV-1 genotypes in CSF and plasma and analysis of related factors. Virulence, 2023.

A104. Yu et al. Inflammatory/immune-suppressive microenvironment characteristics of pancreatic cancer patients with Shi-Re syndrome identified by extracellular vesicle long RNA profiling. Traditional Medicine Research, 2023.

A105. Wang et al. Exosome-transported lncRNA H19 regulates insulin-like growth factor-1 via the H19/let-7a/insulin-like growth factor-1 receptor axis in ischemic stroke. Neural Regeneration Research, 2023.

A106. Wang et al. Characterization of exogenous sequence fragments in extracellular vesicles from human. Small structures, 2023.

A107. Bersani et al. Exploring circular MET RNA as a potential biomarker in tumors exhibiting high MET activity. Journal of Experimental & Clinical Cancer Research, 2023.

A108. Simushi et al. Verification of dried blood spot as a sample type for HIV viral load and early infant diagnosis on Hologic Panther in Zambia. BMC Research Notes, 2023.

A109. Khan et al. Characterization of rare spontaneous human immunodeficiency virus viral controllers attending a national United Kingdom clinical service using a combination of serology and molecular diagnostic assays. Open Forum Infectious Diseases, 2023.

A110. Chen et al. Multi-omics profiling reveals potential alterations in rheumatoid arthritis with different disease activity levels. Arthritis Research & Therapy, 2023.

A111. Timofeeva et al. Prediction of early- and late-onset pre-eclampsia in the preclinical stage via placenta-specific extracellular miRNA profiling. International Journal of Molecular Sciences, 2023.

A112. Lambert-Niclot et al. Four days/week antiretroviral maintenance strategy (ANRS 170 QUATUOR): substudies of reservoirs and ultrasensitive drug resistance. Journal of antimicrobial Chemotherapy, 2023.

A113. Song et al. Differential expression of exosomal miRNAs and proteins in the plasma of systemic lupus erythematous patients. Heliyon, 2023.

A114. Lu et al. The potential of tRF-21-U0EZY9X1B plasmatic level as a biomarker of children with obstructive sleep apnea-hypopnea syndrome. BMC Pediatrics, 2023.

A115. Li et al. Circulating extracellular vesicles are associated with the clinical outcomes of sepsis. Frontiers in Immunology, 2023.

A116. Pei et al. The diagnostic combination of serum circulating miR-488 and lncRNA AC018761 as biomarkers for hypopharyngeal squamous cell carcinoma (HPSCC). Arabian Journal of Chemistry, 2023.

A117. Guo et al. Gastric cancer-associated long non-coding RNA profiling and noninvasive biomarker screening based on a high-risk population cohort. Cancer Medicine, 2023.

A118. Fourgeaud et al. Chronic Aichi virus infection as a cause of long-lasting multiorgan involvement in patients with primary immune deficiencies. Clinical Infectious Diseases, 2023.

A119. Zhuang et al. Circular RNA COL1A2 mediates high glucose-induced oxidative stress and pyroptosis by regulating miR-424-5p/SGK1 in diabetic nephropathy. Applied Biochemistry and Biotechnology, 2023.

A120. Liao et al. Plasma extracellular vesicle transcriptomics identifies CD160 for predicting immunochemotherapy efficacy in lung cancer. Cancer Science, 2023.

A121. Vardaki et al. Transcriptomic analysis of plasma exosomes provides molecular information of response to cabazitaxel treatment in men with metastatic castration-resistant prostate cancer. Prostate, 2023.

A122. Wang et al. CircRNA circSLIT2 is a novel diagnostic and prognostic biomarker for gastric cancer. Wiener Klinische Wochenschrift, 2023.

A123. Hu et al. Assessing breast cancer molecular subtypes using extracellular vesicles mRNA. Analytical Chemistry, 2023.

A124. Fratantonio et al. The RNA cargo in small extracellular vesicles from chicken eggs is bioactive in C57BL/6 J mice and human peripheral blood mononuclear cells ex vivo. Frontiers in Nutrition, 2023.

A125. Jin et al. Identification of novel cell-free RNAs in maternal plasma as preterm biomarkers in combination with placental RNA profiles. Journal of Translational Medicine, 2023.

A126. Lai et al. CircFAM114A2 inhibits the progression of hepatocellular carcinoma via miR-630/HHIP axis. Cancer Medicine, 2023.

A127. Hlavay et al. Human pegivirus viremia in HCV/HIV co-infected patients: direct acting antivirals exert anti-pegivirus effects. Journal of Clinical Virology, 2023.

A128. Cao et al. Prevalence of primary drug resistance among newly diagnosed HIV-1-infected individuals in hunan province, China. Aids Research and Human Retroviruses, 2023.

A129. Tang et al. Tumor cells-derived exosomal circVCP promoted the progression of colorectal cancer by regulating macrophage M1/M2 polarization. Gene, 2023.

A130. Jeewanraj et al. Partial compartmentalisation of HIV-1 subtype C between lymph nodes, peripheral blood mononuclear cells and plasma. Virology, 2023.

A131. Singh et al. RNA profile of immuno-magnetically enriched lung cancer associated exosomes isolated from clinical samples. Cancer Genetics, 2023.

A132. Papadimitriou et al. miRNA-seq identification and clinical validation of CD138+ and circulating miR-25 in treatment response of multiple myeloma. Journal of Translational Medicine, 2023.

A133. Li et al. Plasma exosomes from patients with acute myocardial infarction alleviate myocardial injury by inhibiting ferroptosis through miR-26b-5p/ SLC7A11 axis. Life Sciences, 2023.

A134. Jin et al. Cell-free circulating tumor RNAs in plasma as the potential prognostic biomarkers in colorectal cancer. Frontiers in Oncology, 2023.

A135. Harada et al. Circulating miR-20b-5p and miR-330-3p are novel biomarkers for progression of atrial fibrillation: Intracardiac/extracardiac plasma sample analysis by small RNA sequencing. Plos One, 2023.

A136. Abdallah et al. Expression signature of immune-related microRNAs in autoimmune skin disease: psoriasis and vitiligo insights. Molecular Diagnosis & Therapy, 2023.

A137. Silva et al. Occult hepatitis C infection identified in injection drug users with direct antiviral agents therapy and spontaneous resolution of hepatitis C virus infection. Virus Research, 2023.

A138. Khadka et al. Circulating microRNA biomarker for detecting breast cancer in high-risk benign breast tumors. International Journal of Molecular Sciences, 2023.

A139. Zabegina et al. Diagnosis of prostate cancer through the multi-ligand binding of prostate-derived extracellular vesicles and miRNA analysis. Life-Basel, 2023.

A140. Flores-Chova et al. Plasma exosomal non-coding RNA profile associated with renal damage reveals potential therapeutic targets in lupus nephritis. International Journal of Molecular Sciences, 2023.

A141. Weiner et al. Maternal plasma RNA in first trimester nullipara for the prediction of spontaneous preterm birth <= 32 weeks: validation study. Biomedicines, 2023.

A142. Vicenti et al. SARS-CoV-2 neutralizing antibodies to B.1 and to BA.5 variant after booster dose of BNT162b2 vaccine in HIV patients COVID-naive and on successful antiretroviral therapy. Vaccines, 2023.

A143. Ferreira et al. HIV promotes atherosclerosis via circulating extracellular vesicle microRNAs. International Journal of Molecular Sciences, 2023.

A144. Xu et al. miR-18a and miR-106a signatures in plasma small EVs are promising biomarkers for early detection of pancreatic ductal adenocarcinoma. International Journal of Molecular Sciences, 2023.

A145. Brioschi et al. Impact of Sacubitril/Valsartan on circulating microRNA in patients with heart failure. Biomedicines, 2023.

A146. Orf et al. Metagenomic detection of divergent insect- and bat-associated viruses in plasma from two African individuals enrolled in blood-borne surveillance. Viruses-Basel, 2023.

A147. Zanella et al. Longitudinal detection of twenty DNA and RNA viruses in allogeneic hematopoietic stem cell transplant recipients plasma. Viruses-Basel, 2023.

A148. Gager et al. Expression patterns of miR-125a and miR-223 and their association with diabetes mellitus and survival in patients with non-ST-segment elevation acute coronary syndrome. Biomedicines, 2023.

A149. Lak et al. Cell-Free RNA from plasma in patients with neuroblastoma: exploring the technical and clinical potential. Cancers, 2023.

A150. Raccagni et al. HIV viral load monitoring during monkeypox virus infection among people with HIV. AIDS, 2023.

A151. Zhang et al. Up-regulated lncRNA SNHG9 mediates the pathogenesis of dilated cardiomyopathy via miR-326/EPHB3 axis. Journal of Thrombosis and Thrombolysis, 2023.

A152. Wang et al. Effect of electroacupuncture on haemodynamic changes during intubation for general anaesthesia is mediated by nitric oxide synthase-3 via the regulation of microRNA-155, microRNA-335 and microRNA-383. Molecular Medicine Reports, 2023.

A153. Huang et al. Week 96 results of switching from tenofovir disoproxil fumarate-based antiretroviral therapy to coformulated Elvitegravir, Cobicistat, Emtricitabine, and Tenofovir Alafenamide among HIV/hepatitis B virus-coinfected patients. Microbiology Spectrum, 2023.

A154. Carna et al. Missorting of plasma miRNAs in aging and Alzheimer's disease. Journal of Neurochemistry, 2023.

A155. Xu et al. Hsa_circ_0079598 acts as a potential diagnostic and prognostic biomarker for gastric cancer. Oncologie, 2023.

A156. Yan et al. LINC01234 sponging of the miR-513a-5p/AOX1 axis is upregulated in osteoporosis and regulates osteogenic differentiation of bone marrow mesenchymal stem cells. Molecular Biotechnology, 2023.

A157. Elmoselhi et al. Circulating microRNAs as potential biomarkers of early vascular damage in vitamin D deficiency, obese, and diabetic patients. Plos One, 2023.

A158. Xu et al. Polycyclic aromatic hydrocarbons exposure and plasma lncRNA signature: A profile and functional analysis. Science of the Total Environment, 2023.

A159. Roška et al. Integrated Microarray-Based Data Analysis of miRNA Expression Profiles: Identification of Novel Biomarkers of Cisplatin-Resistance in Testicular Germ Cell Tumours. International Journal of Molecular Sciences, 2023.

A160. Hu et al. Exosome-derived circCCAR1 promotes CD8+T-cell dysfunction and anti-PD1 resistance in hepatocellular carcinoma. Molecular Cancer, 2023.

A161. Li et al. Plasma exosome-derived circGAPVD1 as a potential diagnostic marker for colorectal cancer. Translational Oncology, 2023.

A162. Prins et al. The BAF complex inhibitor pyrimethamine reverses HIV-1 latency in people with HIV-1 on antiretroviral therapy. Science Advances, 2023.

A163. Jia et al. Safety, tolerability, pharmacokinetics, and antiviral activity of the novel core protein allosteric modulator ZM-H1505R (Canocapavir) in chronic hepatitis B patients: a randomized multiple-dose escalation trial. BMC Medicine, 2023.

A164. Garcia-Valiente et al. Understanding repertoire sequencing data through a multiscale computational model of the germinal center. NPJ Systems Biology and Applications, 2023.

A165. Wei et al. Decreased miR-127 promotes the occurrence of breast cancer via increasing the expression of SPP1. Advances in Clinical and Experimental Medicine, 2023.

A166. Ikeda et al. Resistin G-A haplotype at SNP-420/-358 is associated with the latent sarcopenic obesity index in the toon genome study. Journal of Diabetes Investigation, 2023.

A167. Yang et al. lncRNA-TCONS_00008552 expression in patients with pulmonary arterial hypertension due to congenital heart disease. Plos One, 2023.

A168. Tahermanesh et al. Evaluation of expression of biomarkers of PLAGL1 (ZAC1), microRNA, and their non-coding RNAs in patients with endometriosis. Journal of Gynecology Obstetrics and Human Reproduction, 2023.

A169. Henderson et al. Cerebrospinal fluid virology in people with HIV. HIV Medicine, 2023.

A170. Everaert et al. Blocking abundant RNA transcripts by high-affinity oligonucleotides during transcriptome library preparation. Biological Procedures Online, 2023.

A171. Damas et al. One for all, all for one: neuro-HIV multidisciplinary platform for the assessment and management of neurocognitive complaints in people living with HIV. HIV Medicine, 2023.

A172. Yuan et al. CircRNA DICAR as a novel endogenous regulator for diabetic cardiomyopathy and diabetic pyroptosis of cardiomyocytes. Signal Transduction and Targeted Therapy, 2023.

A173. Chen et al. Immune reconstitution inflammatory syndrome in people living with HIV who presented with interstitial pneumonitis: an emerging challenge in the era of rapid initiation of antiretroviral therapy. Microbiology Spectrum, 2023.

A174. Srinath et al. Plasma metabolites with mechanistic and clinical links to the neurovascular disease cavernous angioma. Communications Medicine, 2023.

A175. Vallejos et al. Plasma exosome gene signature differentiates colon cancer from healthy controls. Annals of Surgical Oncology, 2023.

A176. Zheng et al. The circRNA-miRNA-mRNA regulatory network in plasma and peripheral blood mononuclear cells and the potential associations with the pathogenesis of systemic lupus erythematosus. Clinical Rheumatology, 2023.

A177. Han et al. Plasma extracellular vesicle messenger RNA profiling identifies prognostic EV signature for non-invasive risk stratification for survival prediction of patients with pancreatic ductal adenocarcinoma. Journal of Hematology and Oncology, 2023.

A178. Eldosoky et al. Diagnostic Significance of hsa-miR-21-5p, hsa-miR-192-5p, hsa-miR-155-5p, hsa-miR-199a-5p Panel and Ratios in Hepatocellular Carcinoma on Top of Liver Cirrhosis in HCV-Infected Patients. International Journal of Molecular Sciences, 2023.

A179. Mei et al. High-resolution genomic profiling of a genotype 3b hepatitis C virus from a flare of an occult hepatitis patient with acute-on-chronic liver failure. Viruses-Basel, 2023.

A180. Wang et al. Downregulated circulating long non-coding RNA GAS6-AS1 screens and predicts acute myocardial infarction. Anatolian Journal of Cardiology, 2023.

A181. Levstek et al. Interplay between microRNAs, serum proprotein convertase subtilisin/kexin type 9 (PCSK9), and lipid parameters in patients with very high lipoprotein(a) treated with PCSK9 inhibitors. Genes, 2023.

A182. Mehta et al. Pharmacokinetic and pharmacokinetic/pharmacodynamic characterization of the dolutegravir/rilpivirine two-drug regimen in SWORD-1/-2 phase 3 studies. British Journal of Clinical Pharmacology, 2023.

A183. Liu et al. Effect of miR-29a-3p in exosomes on glioma cells by regulating the PI3K/AKT/HIF-1 alpha pathway. Molecular Medicine Reports, 2023.

A184. Gilyazova et al. Exosomal miRNA-155 and miRNA-146a are promising prognostic biomarkers of the severity of hemorrhagic fever with renal syndrome. Non-coding RNA Research, 2023.

A185. Liang et al. A plasma 3-marker microRNA biosignature distinguishes spinal tuberculosis from other spinal destructive diseases and pulmonary tuberculosis. Frontiers in Cellular and Infection Microbiology, 2023.

A186. Li et al. Bioinspired CRISPR-mediated cascade reaction biosensor for molecular detection of HIV using a glucose meter. ACS Nano, 2023.

A187. Ye et al. LncGMDS-AS1 promotes the tumorigenesis of colorectal cancer through HuR-STAT3/Wnt axis. Cell Death & Disease, 2023.

A188. Yang et al. CircRNA_0075723 protects against pneumonia-induced sepsis through inhibiting macrophage pyroptosis by sponging miR-155-5p and regulating SHIP1 expression. Frontiers in Immunology, 2023.

A189. Saha et al. Analysis of neurotransmitters validates the importance of the dopaminergic system in autism spectrum disorder. World Journal of Pediatrics, 2023.

A190. Jiang et al. Plasma long noncoding RNAs lncDC and THRIL as potential diagnostic markers of adult primary immune thrombocytopenia. International Journal of Laboratory Hematology, 2023.

A191. Tofukuji et al. Safety and effectiveness analyses of Dolutegravir/Lamivudine in patients with HIV: 2-year report of post-marketing surveillance in Japan. Advances in Therapy, 2023.

A192. Lai et al. CEBPB promotes gastrointestinal motility dysfunction after severe acute pancreatitis via the MALAT1/CIRBP/ERK axis. Molecular Immunology, 2023.

A193. Bajo-Santos et al. Plasma and urinary extracellular vesicles as a source of RNA biomarkers for prostate cancer in liquid biopsies. Frontiers in Molecular Biosciences, 2023.

A194. Sarshari et al. Quantitative analysis of Epstein-Barr virus DNA in plasma and stomach biopsies of patients with gastric cancer. Virus Genes, 2023.

A195. Marin et al. Immune recovery among Romanian HIV/AIDS patients receiving darunavir/ritonavir or darunavir/cobicistat regimens in cART management: A three-year study. Biomedicine & Pharmacotherapy, 2023.

A196. Crisinel et al. Successful implementation of new Swiss recommendations on breastfeeding of infants born to women living with HIV. European Journal of Obstetrics & Gynecology and Reproductive Biology, 2023.

A197. Wang et al. The predictive value of plasma exosomal lncRNAs/mRNAs in NSCLC patients receiving immunotherapy. Advances in Medical Sciences, 2023.

A198. Sun et al. Identification of lncRNA-miRNA-mRNA networks in circulating exosomes as potential biomarkers for systemic sclerosis. Frontiers in Medicine, 2023.

A199. Zhang et al. Hypoxic glioma cell-secreted exosomal circ101491 promotes the progression of glioma by regulating miR-125b-5p/EDN1. Brain Research Bulletin, 2023.

A200. Lopez et al. Patients with abdominal aortic aneurysms have reduced levels of microRNA 122-5p in circulating exosomes. Plos One, 2023.
